# Sugarcane straw returning is an approaching technique for the improvement of rhizosphere soil functionality, microbial community, and yield of different sugarcane cultivars

**DOI:** 10.3389/fmicb.2023.1133973

**Published:** 2023-03-14

**Authors:** Mengrong Wang, Xiaohang Qi, Yujie Shi, Junyang Zhao, Shakeel Ahmad, Kashif Akhtar, Baoshan Chen, Tengxiang Lian, Bing He, Ronghui Wen

**Affiliations:** ^1^State Key Laboratory for Conservation and Utilization of Subtropical Agro-bioresources, College of Life Science and Technology, Ministry and Province Co-sponsored Collaborative Innovation Center for Sugarcane and Sugar Industry, Guangxi Key Laboratory of Sugarcane Biology, Guangxi University, Nanning, China; ^2^Guangxi Key Laboratory of Agro-Environment and Agric-Products Safety, College of Agriculture, Guangxi University, Nanning, China; ^3^The Key Laboratory of Plant Molecular Breeding of Guangdong Province, College of Agriculture, South China Agricultural University, Guangzhou, China

**Keywords:** bacterial community, fungal community, rhizosphere soil, straw retention, sugarcane cultivar

## Abstract

Sugarcane straw returned to the field has rapidly increased due to the bane on straw burning in China. Straw returning of new sugarcane cultivars has been practiced in the fields. Still, its response has not been explored on soil functionality, microbial community and yield of different sugarcane cultivars. Therefore, a comparison was made between an old sugarcane cultivar ROC22 and a new sugarcane cultivar Zhongzhe9 (Z9). The experimental treatments were: without (R, Z), with straw of the same cultivar (RR, ZZ), and with straw of different cultivars (RZ, ZR). Straw returning improved the contents of soil total nitrogen (TN by 73.21%), nitrate nitrogen (NO_3_^—^N by 119.61%), soil organic carbon (SOC by 20.16%), and available potassium (AK by 90.65%) at the jointing stage and were not significant at the seedling stage. The contents of NO_3_^—^N was 31.94 and 29.58%, available phosphorus (AP 53.21 and 27.19%), and available potassium (AK 42.43 and 11.92%) in RR and ZZ were more than in RZ and ZR. Straw returning with the same cultivar (RR, ZZ) significantly increased the richness and diversity of the rhizosphere microbial community. The microbial diversity of cultivar Z9 (treatment Z) was greater than that of cultivar ROC22 (Treatment R). In the rhizosphere, the relative abundance of beneficial microorganisms *Gemmatimonadaceae, Trechispora, Streptomyces, Chaetomium,* etc., increased after the straw returned. Sugarcane straw enhanced the activity of *Pseudomonas* and *Aspergillus* and thus increased the yield of sugarcane., The richness and diversity of the rhizosphere microbial community of Z9 increased at maturity. In ROC22, bacterial diversity increased, and fungal diversity decreased. These findings collectively suggested that the impact of Z9 straw returning was more beneficial than ROC22 on the activity of rhizosphere microorganism’s soil functionality and sugarcane production.

## 1. Introduction

The sugarcane area in Guangxi Province is about 800,000 hectares and is 60% of the sugarcane planting area in China (Zhang and Govindaraju, 2018). Recently, the banon straw burning has been strictly enforced as a legal provision (Fang et al., 2020). The resource utilization of sugarcane straw, such as power generation, feed, and organic fertilizer, has not been commercialized, and sugarcane straw returning has been rapidly popularized in a short time. Sugarcane straw can reach 6–24 Mg ha^−1^ and contains many nutrients (N, P, K, etc.) (Cherubin et al., 2018; White et al., 2018). The straw’s nutrients were immobilized. Thus, the decomposition of straw provides carbon (C) and nitrogen (N) to the soil (de Abreu Sousa Junior et al., 2018). Straw decomposition is a complex process and is predominantly mediated by soil microorganisms with specialized functions (Yang et al., 2022). The microbial community is an essential part of the environment, and plant-microbe interaction affects the function of the ecosystem (Vivanco et al., 2018; Morais et al., 2019). Soil rhizosphere microbial diversity might increase nutrient availability and plant productivity (Alawiye and Babalola, 2019; Sardar et al., 2021a). Many microorganisms play important roles in plant-litter decomposition and release of soil organic matter (Yarwood, 2018). Beneficial soil microorganisms also contribute to pathogen resistance (Ghorbanpour et al., 2018), plant nutrition, and growth (Smith and Smith, 2012). In turn, plants can also provide nutrients to rhizosphere bacteria by secreting carbon metabolites through their roots (Bunemann et al., 2018).

In addition, soil microbial communities and nutrient cycling are usually affected by the interactions of agricultural management (e.g., straw return) and the rhizosphere environment (Sardar et al., 2021b; Zhao et al., 2022). Straw return is a common agricultural management technique used to increase soil fertility and soil organic carbon (Castioni et al., 2018; Lisboa et al., 2018). With the development of sequencing technology, growing research has been conducted on the impact of sugarcane straw on soil microorganisms. Zhang C. et al. (2019) demonstrated the effects of sugarcane straw retention on soil bacteria at different depths (Franco et al., 2013). They found that sugarcane straw retention enriched bacteria taxa related to lignocellulose decomposition and nutrient cycling (C and N). Suleiman et al. (2018) studied the effects of straw, combined with nitrogen fertilizer (or vinasse), on the structure and function of soil microbial communities. The results showed that microorganisms related to carbon compound degradation and nitrogen metabolism increased significantly after adding straw. Plant genotype can affect the related microorganisms (Aira et al., 2010; Wagner et al., 2016). Soybean varieties with different resistances may change the composition of the rhizosphere microbial community and regulate the presence of beneficial microbial community, and improve the growth and defensive mechanisms of plant against pathogen invasion (Mendes et al., 2018). Wu et al. (2016) validated the significant influence of mangrove species on the rhizosphere bacterial community by evaluating the changes in three types of mangrove trees. The maize rhizosphere microbial community structure was significantly affected by maize genotypes (Aira et al., 2010). However, for different sugarcane varieties, the effect of straw returning on yield and rhizosphere microorganisms is still unknown and needs work.

ROC22 is a cultivar that has lasted for 30 years in China; its planting area has remained first place for a long time (Wei, 2012). Long-term cultivation of a single variety has led to an increase in serious diseases, and the yield has decreased to farmers’ expectations (Wei, 2012). Recently, some new sugarcane varieties have been bred and gradually replaced ROC22. Z9 is a newly cultivated variety with a high yield and short planting history, which was selected from the ROC25 × Yunzhe 89–7 combination (Zhang S. S. et al., 2019). When ROC22 are harvested, new sugarcane varieties are planted, and straw returning with different plant variety occurs. Due to the differences in the nutritional components and elements in sugarcane leaves, the effects of straw with an another distinct cultivar on sugarcane yield and rhizosphere microbial community structure are unknown.

Researchers have worked on the performance of similar filed straw returning, but the key point is that the response of different cultivars (fields) straws on the different sugarcane cultivars has not been studied that how straw of the different cultivars performed in the similar or different field, therefore, Sugarcane cultivars Z9 and ROC22 were selected to compare the effects of straw returning on soil properties, rhizosphere microbial community structure, and yield of sugarcane cultivars. In general, straw returning improves the soil properties, potentially increasing yield and significantly improving the diversity of rhizosphere microbial communities. Straw returning with the same variety significantly increased the rhizosphere microbial community richness and diversity than a different cultivar’s straw returning. High-yielding new variety Z9 can induce the diversity of rhizosphere microbial community more effectively with and without straw returning than the old variety ROC22.

## 2. Materials and methods

### 2.1. Site description and experimental design

The two sugarcane cultivars used in this study were Zhongzhe9 (Z9) and ROC22. The total nitrogen, phosphorus, and potassium contents in Z were higher than in ROC22. The ROC22 cultivar has a long planting history and is the largest planting area in China. In contrast, Z9 is a newly cultivated variety with a high yield and short planting history developed by the State Key Laboratory of Conservation and Utilization of Subtropical Agro-bioresources.

The experiment was designed and conducted from September 2019 to August 2020 at the green-house located at Fusui, Chongzuo, Guangxi province, China (N 22°30′48″, E 107°46′28″). The greenhouse has a total area of 2,000 m^2^, is equipped with ventilation and water spraying devices, and has a transparent roof. The experimental field of sugarcane was subjected to the following treatments: (I) ROC22 without straw (R); (II) cultivar Z9 without straw (Z); (III) cultivar ROC22 plus ROC22 straw (RR); (IV) cultivar Z9 plus Z9 straw (ZZ); (V) cultivar ROC22 plus Z9 straw (RZ); and (VI) cultivar Z9 plus ROC22 straw (ZR); with three replicates per treatment. The experiment had a random block design with 18 blocks, with each block covering an area of 5 × 6 m. Each plot had six rows of sugar cane, with a total of 200 buds. The distance between two rows of sugarcane was 1.2 m, and the distance between two blocks was 2 m. Two cultivars of sugarcane straw (including the tips) were collected from the field and crushed separately into approximately 3–5 cm fragments with a pulverizer. According to the appropriate treatment, once the sugarcane was planted, the straw was evenly distributed at a dry weight of 12 Mg ha^−1^ (Lian et al., 2019; Pimentel et al., 2019). The physicochemical properties of soil and sugarcane straw used in this experiment were as follows: (i) soil: lateritic red earth, pH of 5.66, 15.46 g/kg soil organic carbon (SOC), 26.66 g/kg organic matter (OM), 1.29 g/kg total nitrogen (TN), 0.61 mg/kg ammonium nitrogen (NH_4_^+^-N), 56.32 mg/kg nitrate nitrogen (NO_3_^−^-N), 73.29 mg/kg available phosphorus (AP), and 114.80 mg/kg available potassium (AK); (ii) sugarcane straw of ROC22: 43.40% total carbon (TC), 1.20% total nitrogen (TN), and C:N ratio 36.32; (iii) sugarcane straw of Z 9: 41.84% TC, 1.45% TN, and C:N ratio 28.79. The annual average temperature of the screen house was 28°C, the highest temperature was 40°C, and the lowest temperature was 2°C. These two cultivars were grown on the same experimental field, and regular ventilation and irrigation were performed in the greenhouse to keep the soil moisture content in the 80 ± 5% range. Fertilizing and soil cultivating took place according to regular sugarcane planting management techniques. At the same time, the sugarcane straw was mechanically turned into soil.

### 2.2. Soil sample collection and physicochemical analysis

The samples were collected on May 20, 2020, at the jointing stage of sugarcane growth. Each block adopted a multi-point sampling method to collect sugarcane rhizosphere soil, which was mixed to form a sample. After the sugarcane roots were dug out, larger clods were removed, and then the roots were placed into a clean bag and shaken vigorously (Lian et al., 2019). Two samples were taken from each plot, and each sample consisted of the rhizosphere soil from five random sugarcanes. Thirty-two soil samples were collected from eighteen blocks. Approximately two grams of soil were placed into a sterilized 2-ml centrifuge tube and stored at −80°C for DNA extraction. Part of the remaining soil was stored at 4°C to determine soil ammonium nitrogen and nitrate nitrogen. The remainder of the soil was air-dried at room temperature (30 to 35°C) to measure the physicochemical properties of the soil.

The soil pH was examined in a soil–water slurry (1:2.5 w: v) using a potentiometer with a pH meter (FE20, Mettler Toledo, Germany). The volumetric method was used to measure the content of soil organic carbon (SOC) and organic matter (OM). Ammonium nitrogen (NH_4_^+^-N), nitrate nitrogen (NO_3_^−^-N), and available phosphorus (AP) were also detected by the kit (Beijing Solarbio Science & Technology Co., Ltd., China). Available potassium (AK) was determined using inductively coupled plasma-atomic emission spectrometry (ICPS-7510, Shimadzu, Japan). A continuous flow analytical system detected total nitrogen (TN) (AA3, Bran+Luebbe, Germany). The sugarcane yield was measured on August 10, 2020.

### 2.3. DNA extraction and sequencing

A total of 36 DNA samples were extracted using the FastDNA™ spin kit for soil (MP Biomedicals, US) following the manufacturer’s instructions. The DNA concentration was measured using NanoDrop 2000 (Thermo Fisher Scientific, Wilmington, DE, United States), and the quality of PCR products was detected by 1% agarose gel electrophoresis. The V3–V4 region of the 16S rRNA gene was amplified with primer pairs 515F (GTGCCAGCMGCCGCGG) and 907R (CCGTCAATTCMTTTRAGTTT). The primer pair ITS1F (CTTGGTCATTTAGAGGAAGTAA) and ITS2R (GCTGCGTTCTTCATCGATGC) was used to amplify the ITS1 region of fungi. The PCR and sequencing processes were performed by Majorbio Bio-Pharm Technology Co. Ltd. (Shanghai, China) using the Illumina MiSeq PE300 platform. The data were analyzed on the Majorbio Cloud Platform.^1^ The low-quality sequences with a length of less than 200 bp and an average quality score of less than 20 were filtered. OTU clustering was performed on non-repetitive sequences (excluding single sequences) using the Ribosomal Database Project (RDP) Classifier (Lian et al., 2019) as per 97% similarity. The chimera was removed during the clustering process, and the representative sequence of OTU was obtained. The OTUs of bacteria and fungi were analyzed using SILVA (version 132)^2^ and UNITE (version 8.0) databases, respectively. The OTU table was randomly resampled with the smallest sequence number in the samples (28,650 for bacteria and 60,205 for fungi) to assure the same sequencing depth for each sample, and the resampled OTU table was used for a correct comparison between samples. All sequences have been deposited into the NCBI archive under Bio Project ID PRJNA664481.

### 2.4. Statistical and bioinformatics analysis

Perform microbial data (6 plots × 3 repetitions) calculation on the Majorbio cloud platform.^3^ The PCoA analysis, Alpha diversity indices, ANOSIM, Kruskal-Wallis H test, and Wilcoxon rank-sum test analysis to explore the changes in the sugarcane rhizosphere microbial community induced by straw returning. The correlation between environmental factors and microbial community through distance-based redundancy analysis (dbRDA) based on the Bray-Curtis distance algorithm. The correlation analysis of phylum-level microorganisms with environmental factors and functional prediction pathways was analyzed by Cytoscape (3.9.1). A one-way analysis of variance, a significance test between groups, and the normal distribution of physicochemical properties were tested by SPSS19.

## 3. Results

### 3.1. Physicochemical properties of soil and yield of sugarcane

**Figure 1:**
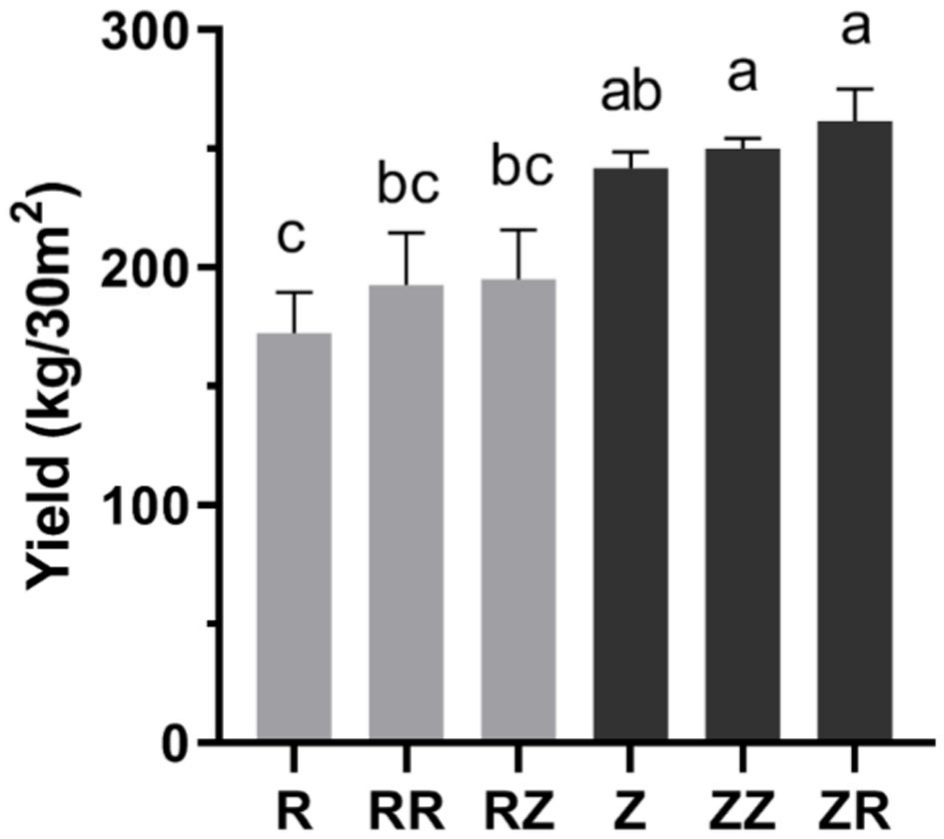
Response of sugarcane straw returning on the yield of different sugarcane cultivars. R: cultivar ROC22, RR: cultivar ROC22 plus straw of ROC22; RZ: cultivar ROC22 plus straw of Zhongzhe9; Z: cultivar Zhongzhe9; ZZ: cultivar Zhongzhe9 plus straw of Zhognzhe9; ZR: cultivar Zhongzhe9 plus straw of ROC22. The same letter of data in each column means no significant difference (*p* > 0.05), while different letters mean a significant difference (*p* < 0.05).

The response of soil physicochemical characteristics under different straw mulch were presented in Table 1. straw of RR, RZ, ZZ, and ZR significantly (*p* < 0.05) enhanced the characteristics of rhizosphere soil, such as SOC, OM, NO_3_^−^-N, and AK, respectively (Table 2). There were also changes in other stage, but the response was different within each variety (Supplementary Tables S1 and S2). Compared with the control without straw (R and Z), total nitrogen content did not significantly differ in the RR but increased significantly in the others. Straw returning significantly decreased the content of ammonium nitrogen. However, with and without straw amendments have no consistent changes in pH (Table 2). Interestingly, although adding sugarcane straw significantly (*p* < 0.05) increased some characteristics, depends on the different cultivar straw amendements. The SOC and OM were higher in RZ and ZR than in RR and ZZ. Contrastingly, NO_3_^−^-N, AP, AK, and pH were higher in RR and ZZ than in RZ and ZR. The sugarcane yield was enhanced in the treatments with straw (Figure 1), although there was no significant difference between RR and RZ or ZZ and ZR. The overall yield of Z9 was higher than ROC22.

**Table 1:** The composition of leaves of different cultivar.

Variety	Phosphorus (g kg^−1^ DM)	Nitrogen (g kg^−1^ DM)	Potassium (g kg^−1^ DM)	Calcium (g kg^−1^ DM)	Magnesium (g kg^−1^ DM)	Sulfur (g kg^−1^ DM)	Crude ash (g/kg DM)	Organics (g/kg DM)	DM (g/kg FM)
ROC22	0.56 ± 0.04	4.80 ± 0.54	3.57 ± 0.36	3.26 ± 0.61	2.16 ± 0.26	0.69 ± 0.05	67.23 ± 4.52	932.77 ± 45.85	328.56 ± 21.68
Zhongzhe9	0.77 ± 0.08	3.11 ± 0.26	4.01 ± 0.29	3.13 ± 0.53	2.56 ± 0.28	0.58 ± 0.07	72.29 ± 6.11	927.71 ± 31.62	359.23 ± 29.25

**Table 2:** Soil characteristics of different treatments with straw.

Treatment	SOC (g kg^−1^)	OM (g kg^−1^)	TN (g kg^−1^)	NH_4_^+^-N (mg kg^−1^)	NO_3_^−^-N (mg kg^−1^)	AP (mg kg^−1^)	AK (mg kg^−1^)	pH (1:2.5)
R	14.53 ± 0.22c	25.06 ± 0.39c	0.94 ± 0.06d	1.20 ± 0.08b	19.58 ± 3.55e	125.67 ± 4.84b	89.89 ± 0.66e	6.01 ± 0.02c
RR	15.64 ± 0.21b	26.97 ± 0.37b	1.33 ± 0.09bcd	0.84 ± 0.06c	43.00 ± 3.30ab	171.43 ± 10.66a	171.38 ± 0.85a	6.06 ± 0.05c
RZ	17.46 ± 0.50a	30.11 ± 0.87a	1.61 ± 0.15abc	1.10 ± 0.09b	32.59 ± 2.58 cd	111.89 ± 5.71b	120.32 ± 0.72d	5.88 ± 0.04d
Z	13.16 ± 0.52d	22.70 ± 0.89d	1.12 ± 0.09 cd	1.53 ± 0.07a	24.83 ± 2.05de	118.59 ± 4.78b	88.68 ± 0.69e	6.72 ± 0.01b
ZZ	15.59 ± 0.38b	26.88 ± 0.66b	1.94 ± 0.30a	1.04 ± 0.08bc	48.92 ± 2.66a	159.80 ± 3.89a	139.33 ± 0.69b	7.03 ± 0.02a
ZR	15.40 ± 0.17b	28.28 ± 0.29b	1.69 ± 0.18ab	1.00 ± 0.07bc	37.75 ± 1.71bc	125.63 ± 2.92b	124.49 ± 0.49c	6.03 ± 0.04c

R: cultivar ROC22, RR: cultivar ROC22 plus straw of ROC22; RZ: cultivar ROC22 plus straw of Z9; Z: cultivar Z9; ZZ: cultivar Z9 plus straw of Zhognzhe9; ZR: cultivar Z9 plus straw of ROC22. OM = SOC × 1.724. Values are the means ± SE (*n* = 6).

### 3.2. Diversity of microbial community

A total number of 1,720,693 high-quality, effective sequences and 6,609 OTUs (at 97% similarity level) were obtained from 36 samples using high-throughput sequencing technology to sequence the V4–V5 region of bacteria in the sugarcane rhizosphere soil. All the sequences retrieved from each sample ranged from 350 to 400 base pairs (bp), with an average length of 376.30 bp, and ranged from 41,321 to 58,959 bp. The Chao1, Shannon index, and coverage were calculated to reflect the alpha diversity of bacterial richness and communities (Table 3, and Supplementary Tables S3 and S4). Results showed that RR, RZ, ZZ, and ZR alpha diversity was significantly higher than R and Z (*p* < 0.05). This indicates that adding sugarcane straw increased the abundance of the bacterium in the rhizosphere soil. The Shannon index of the ROC22 cultivars follows the order of RZ > RR > R, while the Shannon index of the Z9 follows the order of ZZ > ZR ≈ Z. Moreover, compared with Z, the Chao1 and Shannon index of R were lower than that of Z, which indicated the diversity of the microbial community of ROC22 was lower than that of Z9.

**Table 3:** Alpha diversity of the bacterial community and fungal community in different treatments with straw.

Treatment	Bacteria			Fungi		
	Chao1	Shannon	Coverage/%	Chao1	Shannon	Coverage/%
R	3539.44 ± 27.94e	6.42 ± 0.01e	96.92 ± 0.02a	898.9 ± 27.12d	3.97 ± 0.06e	99.73 ± 0.02a
RR	4034.74 ± 43.47a	6.50 ± 0.02d	96.44 ± 0.04d	1121.2 ± 22.801b	4.22 ± 0.03d	99.68 ± 0.01b
RZ	3781.69 ± 30.43 cd	6.65 ± 0.01b	96.69 ± 0.03bc	1027.1 ± 21.45c	4.42 ± 0.07c	99.73 ± 0.02a
Z	3712.93 ± 32.11d	6.54 ± 0.01c	96.72 ± 0.03b	1085.4 ± 15.17bc	4.40 ± 0.03c	99.73 ± 0.01a
ZZ	3878.58 ± 43.55bc	6.74 ± 0.03a	96.61 ± 0.04c	1203.5 ± 25.01a	4.60 ± 0.06b	99.67 ± 0.01b
ZR	3972.49 ± 30.57ab	6.54 ± 0.01c	96.43 ± 0.03d	1090.3 ± 28.20bc	4.76 ± 0.06a	99.74 ± 0.02a

Different letters in the same column meant significant difference at 0.05 level.

The ITS1 region of the fungus was sequenced, and a total of 2,547,594 high-quality sequences and 3,000 OTUs were obtained. The sequences obtained from each sample ranged from 61,587 to 69,907, and 81.86% of sequences were between 220 and 280 bp, with an average length of 247.35 bp. Results showed that adding straw significantly increased fungal richness and diversity in ROC22. The Shannon index of the Z9 cultivar follows the order of ZR > ZZ > Z. However, the Chao1 index of ZZ was higher, and no significant difference was observed in the fungi of ZR. Meanwhile, the fungal diversity of R was also lower than that of the Z group. Therefore, adding the sugarcane straw of the same cultivar could increase the community richness (Chao1 index) of a microbial community more than the sugarcane straw of different cultivars, and adding sugarcane straw of different cultivars could increase the diversity (Shannon index) of a microbial community more than sugarcane straw of the same cultivar.

### 3.3. Microbial community composition of different straw treatments

**Figure 2:**
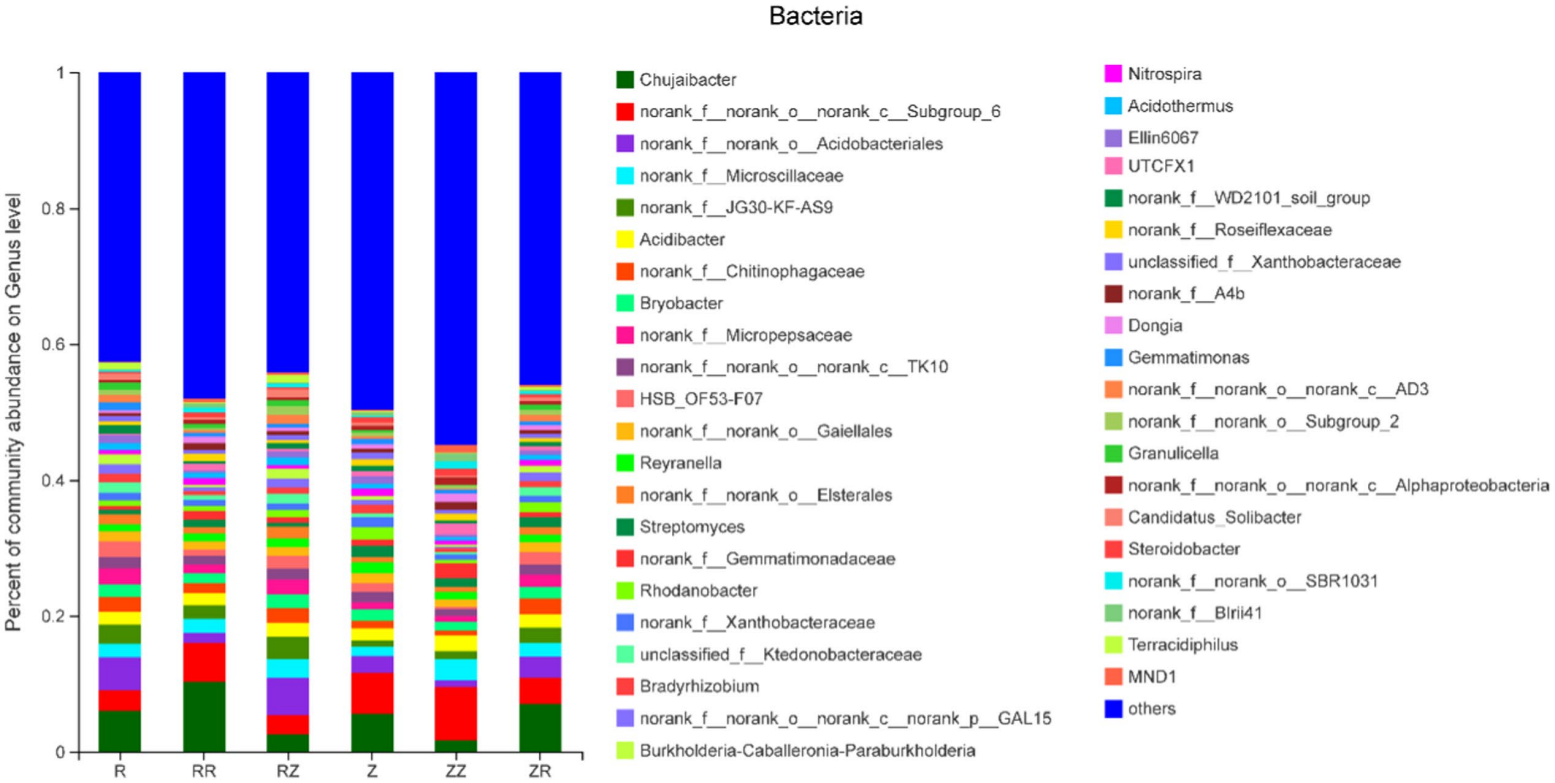
Response of sugarcane straw returning on the relative abundance (% total reads) of bacterial community at the genus level of different sugarcane cultivars R: cultivar ROC22, RR: cultivar ROC22 plus straw of ROC22; RZ: cultivar ROC22 plus straw of Zhongzhe9; Z: cultivar Zhongzhe9; ZZ: cultivar Zhongzhe9 plus straw of Zhognzhe9; ZR: cultivar Zhongzhe9 plus straw of ROC22. ‘Other’ was the sum of genera that relative abundance was lower than 1%.

The rhizosphere soil bacterial sequences were classified into 36 phyla, 105 classes, 280 orders, 312 families, 880 genera, and 1,969 species. The most abundant bacterial phylum was Proteobacteria (34.48~39.82%), followed by Acidobacteria (14.45~18.49%), Chloroflexi (11.02~16.23%), Actinobacteria (9.75~15.83%), and Bacteroidetes (5.73~9.06%) (Figure 2). The proportion of sugarcane rhizosphere bacteria species changed with different treatments while not changing the composition of dominant bacteria, and also, no new phylum was appeared.

Figure 2 shows that different sugarcane straw treatments also had different bacterial communities at the genus level. However, due to different straw types, the abundance of species varied. The most abundant bacteria were Chujaibacter, with an average relative abundance of 5.56%, and this was highest in RR (10.26%). Figure 2 shows that seven of the ten most abundant genera have no clear classification information at the genus level, and the remaining three were Chujaibacter, Acidibacter, and Bryobacter. The rhizosphere soil fungi sequences were classified into 14 phyla, 41 classes, 101 orders, 224 families, 429 genera, and 685 species. The dominant fungi in the phylum were mainly composed of Ascomycota (55.32~79.18%), Basidiomycota (15.06~27.60%), unclassified_k_Fungi (3.75~16.30%), and Mortierellomycota (0.64~1.29%). These four dominant fungi accounted for over 99.7% of sugarcane rhizosphere fungal OTUs (Supplementary Figure S1B). In ROC22, the proportion of *Ascomycota* significantly increased in RR and RZ compared with R. Moreover, the influence of sugarcane straw on the ROC22 fungal community was greater than that of the Z9 variety (Supplementary Figure S1B).

**Figure 3:**
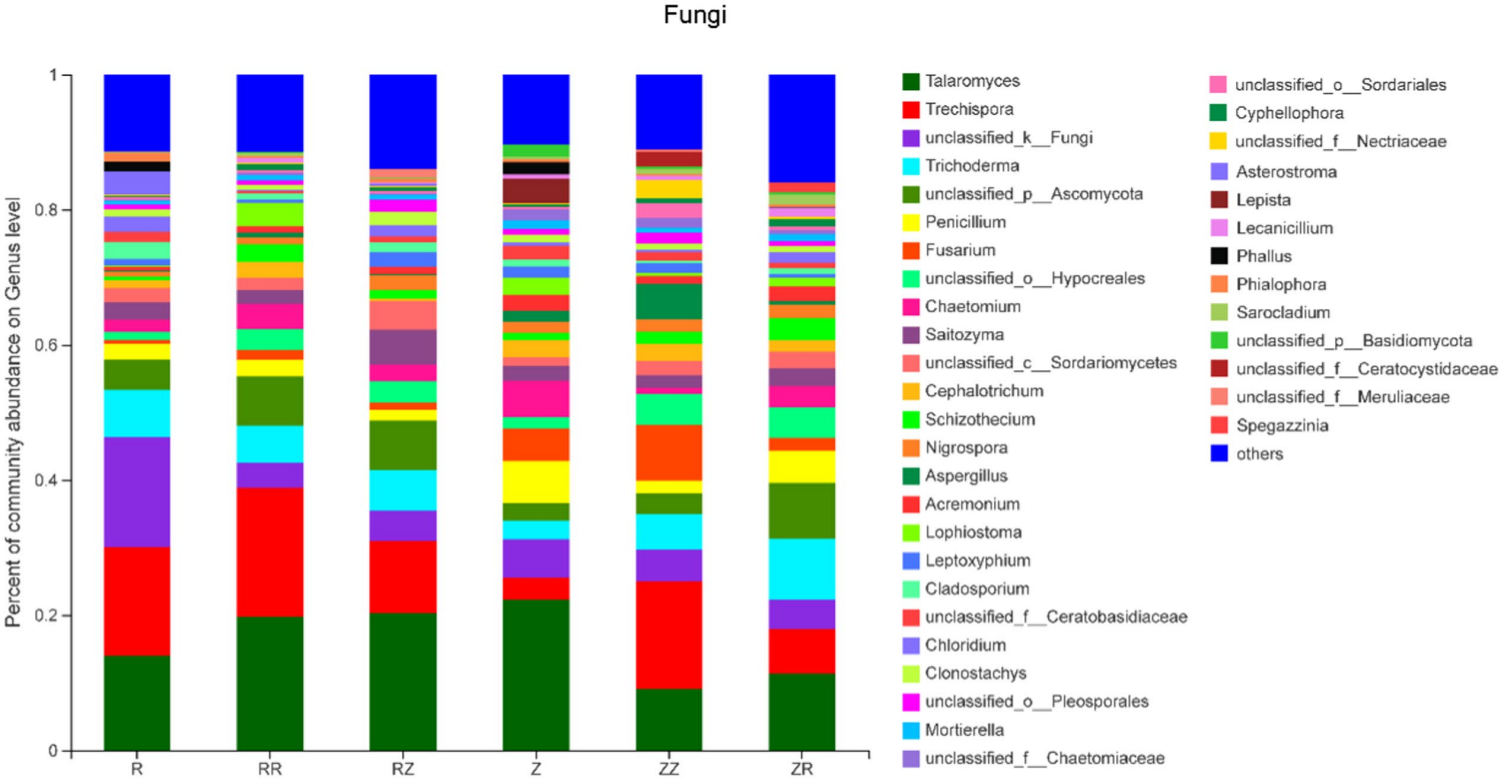
Response of sugarcane straw returning on the relative abundance (% total reads) of fungal community at genus level of different sugarcane cultivars. R: cultivar ROC22, RR: cultivar ROC22 plus straw of ROC22; RZ: cultivar ROC22 plus straw of Zhongzhe9; Z: cultivar Zhongzhe9; ZZ: cultivar Zhongzhe9 plus straw of Zhognzhe9; ZR: cultivar Zhongzhe9 plus straw of ROC22. ‘Other’ was the sum of genera that relative abundance was lower than 1%.

The soil fungal community was dominated by *Talaromyces*, *Trechispora*, *unclassified_k_Fungi*, *Trichoderma*, *unclassified_p_Ascomycota*, and *Penicillium* (Figure 3). It is observable that there are apparent differences in the composition of fungi between the two varieties. The average proportion of the top five fungi in ROC22 accounted for 54%, while in Z9, this was only 38.04%. The average proportion of others shown in Figure 3 was approximately 12.37%, which is significantly lower than that shown in Figure 2. Most of the fungi existed in all samples. However, *Asterostroma* only existed in R and RZ, while lepista only existed in Z. It is noteworthy that *Phallus* only existed in R (1.48%) and Z (1.80%) and disappeared in all treatments with straw (Figure 2; Supplementary Figure S2).

### 3.4. Microbial community structure of different straw treatments

**Figure 4:**
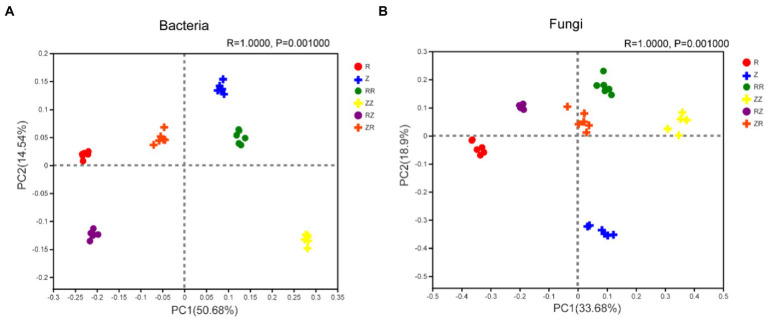
Response of sugarcane straw returning on the Principal Coordinates analysis (PCoA) of bacterial **(A)** and fungal **(B)** community structure of different sugarcane cultivars. R: cultivar ROC22, RR: cultivar ROC22 plus straw of ROC22; RZ: cultivar ROC22 plus straw of Zhongzhe9; Z: cultivar Zhongzhe9; ZZ: cultivar Zhongzhe9 plus straw of Zhognzhe9; ZR: cultivar Zhongzhe9 plus straw of ROC22.R2 value: the degree of explanation of the grouping factor to the sample difference; *p* value less than 0.05 indicates that the reliability of this test is high. PCoA score plot based on Bray-Curtis metrics.

The Principal co-ordinates analysis (PCoA) indicated significant differences in the bacterial communities treated with RR, RZ, ZZ, and ZR compared to the R and Z (Figure 4A; Supplementary Figure S3A). The PC1 and PC2 axes explained 65.22% of the differences in bacterial community between amendments with and without straw returning. The R and *p* values of ANOSIM analyses were 1 and 0.01, respectively. This shows that grouping factors have a high degree of explanation for differences, and the differences between groups are significantly greater than those within groups. PCoA analysis showed that the fungal communities from different treatments were separated (Figure 4B; Supplementary Figure S3B). The PC1 and PC2 axes also explained 52.58% of the differences in fungal communities between amendments with and without straw returning. In addition, bacteria and fungi in the RZ and ZR groups can be distinguished, indicating that different straw cultivars have different effects on the soil microbial community structure (Figure 4). In general, the PCoA results demonstrate that sugarcane species and straw influenced the rhizosphere soil microbial community.

### 3.5. Difference analysis of microbial species

**Figure 5:**
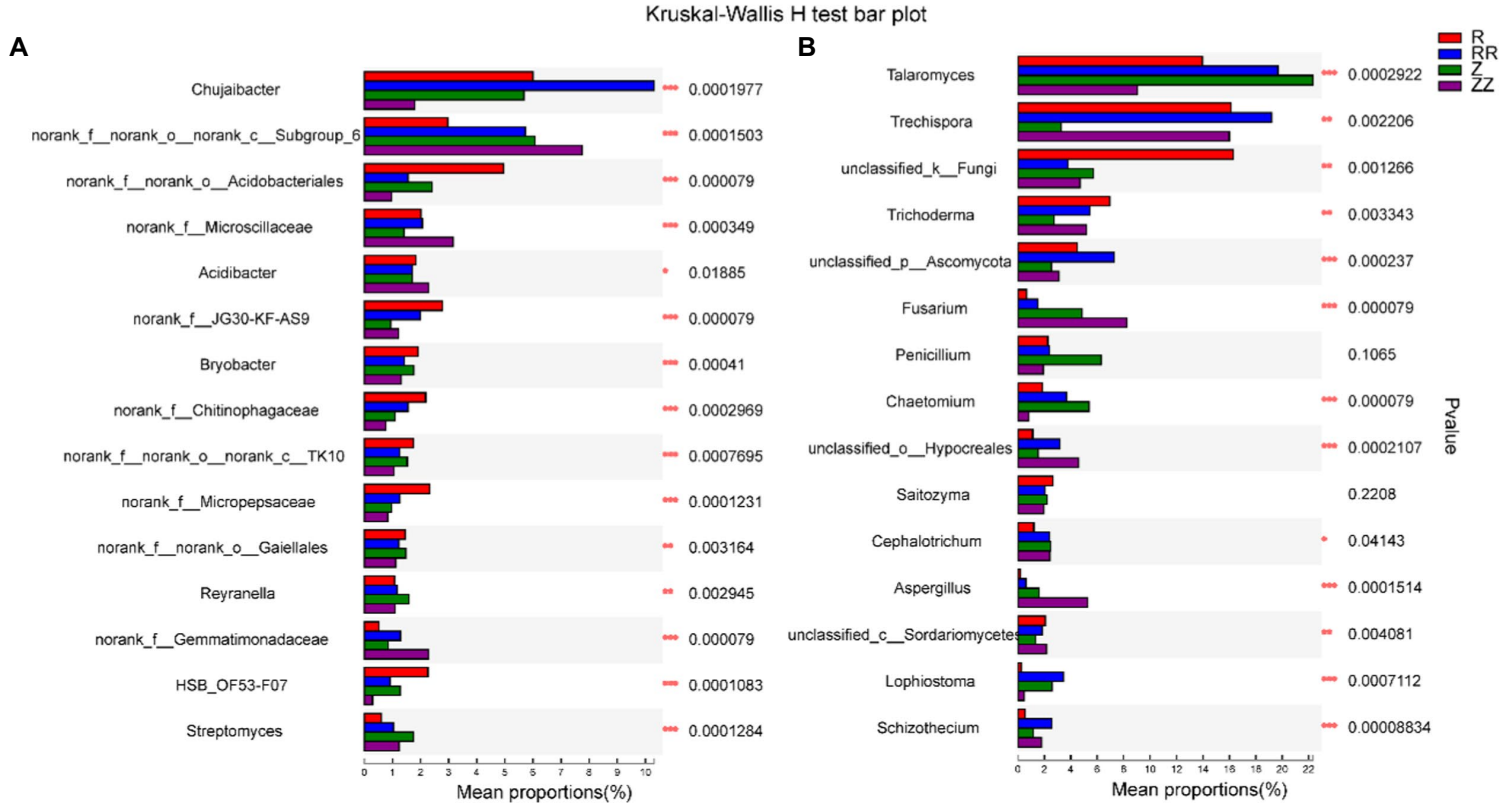
Effect of sugarcane straw returning on the different species analysis of top 15 soil bacterial **(A)** and fungal **(B)** on the genus level of different sugarcane cultivars. The differences between groups were calculated using the Kruskal-Wallis H test. ^∗^*p* < 0.05; ^∗∗^*p* < 0.01.

Figure 5 showed bacteria and fungi with significant differences between the four groups at the genus level using the Kruskal-Wallis rank sum test, listing the 15 most abundant bacteria and fungi. After the sugarcane straw was returned to the field, the same microorganism produced different responses to the straw. For example, *Chujaibacter* and *Talaromyces* increased in the RR group but decreased in the ZZ group. *Trichoderma* decreased in the RR group and increased in the ZZ group. In addition, the relative abundance of genus *Subgroup_6*, *Gemmatimonadaceae*, *Trechispora*, *Fusarium*, and *Hypocreales* was higher in the RR and ZZ. The relative abundance of *Acidobacteriales*, *Bryobacter*, and *Unclassified_k_Fungi* were lower in the RR and ZZ groups.

**Figure 6:**
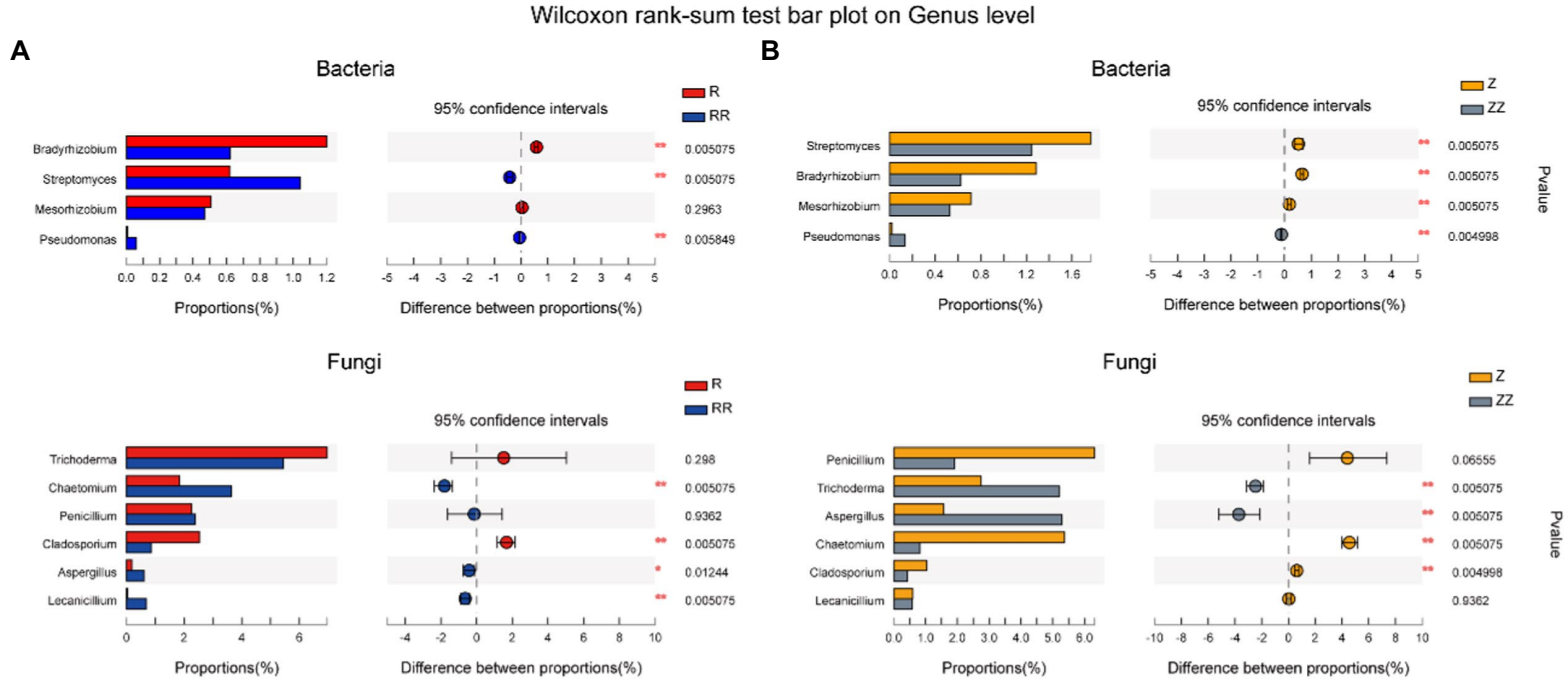
Effect of sugarcane straw returning on the species difference analysis of soil bacterial **(A)** and fungal **(B)** on the genus level of different sugarcane cultivars. The species were related to plant growth. The differences between groups were calculated using Wilcoxon rank-sum test. ^∗^*p* < 0.05; ^∗∗^*p* < 0.01.

To further find the species related to yield, 10 microorganism types related to plant growth were screened out in most abundant 100 microorganisms. Figure 6 shows that *Streptomyces*, *Chaetomium*, *Aspergillus*, and *Lecanicillium* increased significantly in the RR group after amendments with straw (*p* < 0.01), and *Trichoderma* and *Aspergillus* (*p* < 0.01) increased significantly in the ZZ group. Only *Aspergillus* and *Pseudomonas* increased significantly in RR and ZZ compared with R and Z.

### 3.6. Correlation analysis

**Figure 7:**
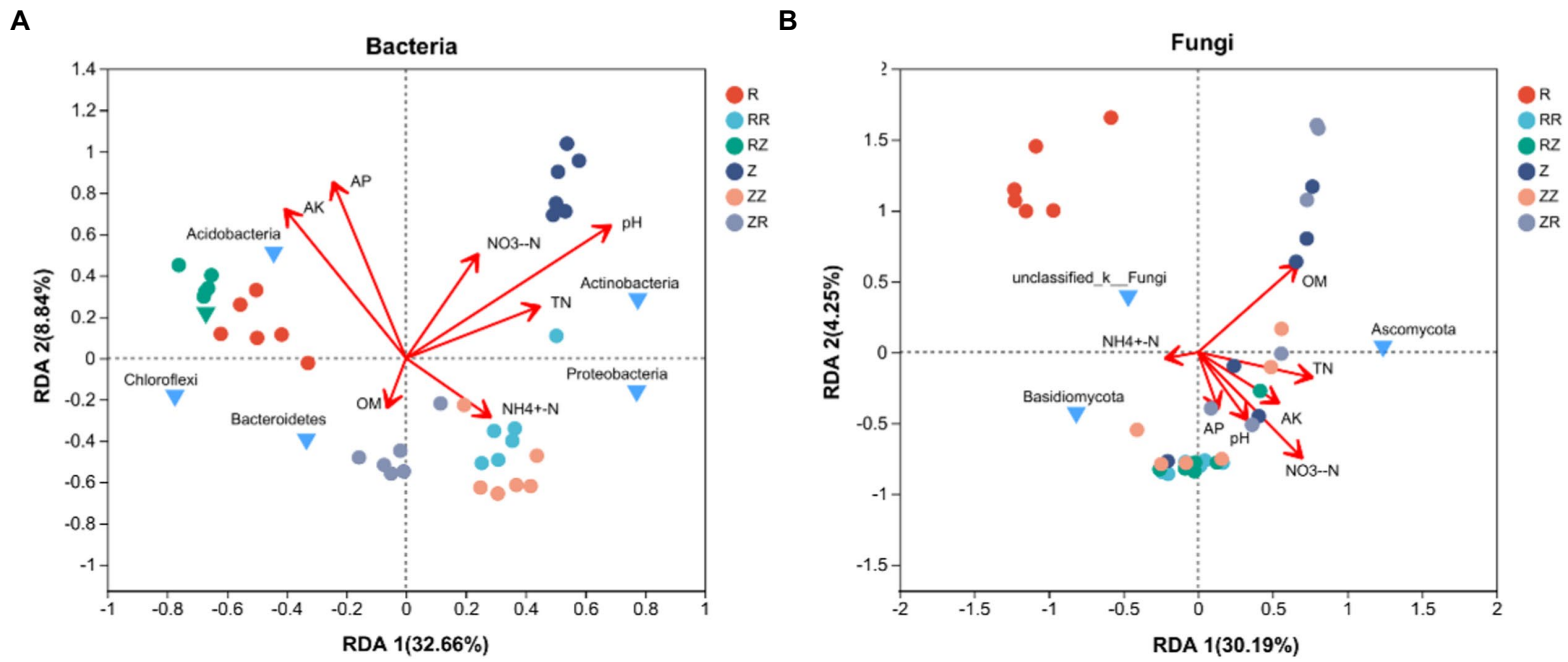
Distance-based redundancy analysis (dbRDA) Redundancy analysis of **(A)** bacteria and **(B)** fungi with environmental factors on different sugarcane cultivars.

We further explored the correlation between environmental factors and microbial community through distance-based redundancy analysis (dbRDA) based on the Bray-Curtis distance algorithm. The two RDA axes explained 32.66 and 8.84% of the total variation, respectively (Figure 7A). AK, AP, and pH are the key environmental factors affecting bacterial community composition at the phylum level. NH_4_^+^-N correlated with the soil bacterial community distribution in RR, ZZ, and ZR groups. Two available nutrients (AK and AP) were correlated with the distribution of the soil bacterial community in groups R and RZ. In contrast, the distribution of the soil bacterial community in group Z was significantly affected by pH. In addition, AK has a strong positive correlation with Acidobacteria, and pH has a strong positive correlation with Actinobacteria. Figure 7B shows the correlation between environmental factors and soil fungal community composition. The two main axes explain 34.44% of fungal community changes in all samples, of which RDA 1 explains 30.19%, and RDA 2 explains 4.25%. OM and NO_3_^−^-N greatly influenced soil fungal community composition in this experiment. Except for NH_4_^+^-N and OM, all the other environmental factors had a strong positive correlation with the soil fungal community in the straw-returning group. In addition, TN had a strong positive correlation with Ascomycota.

**Figure 8:**
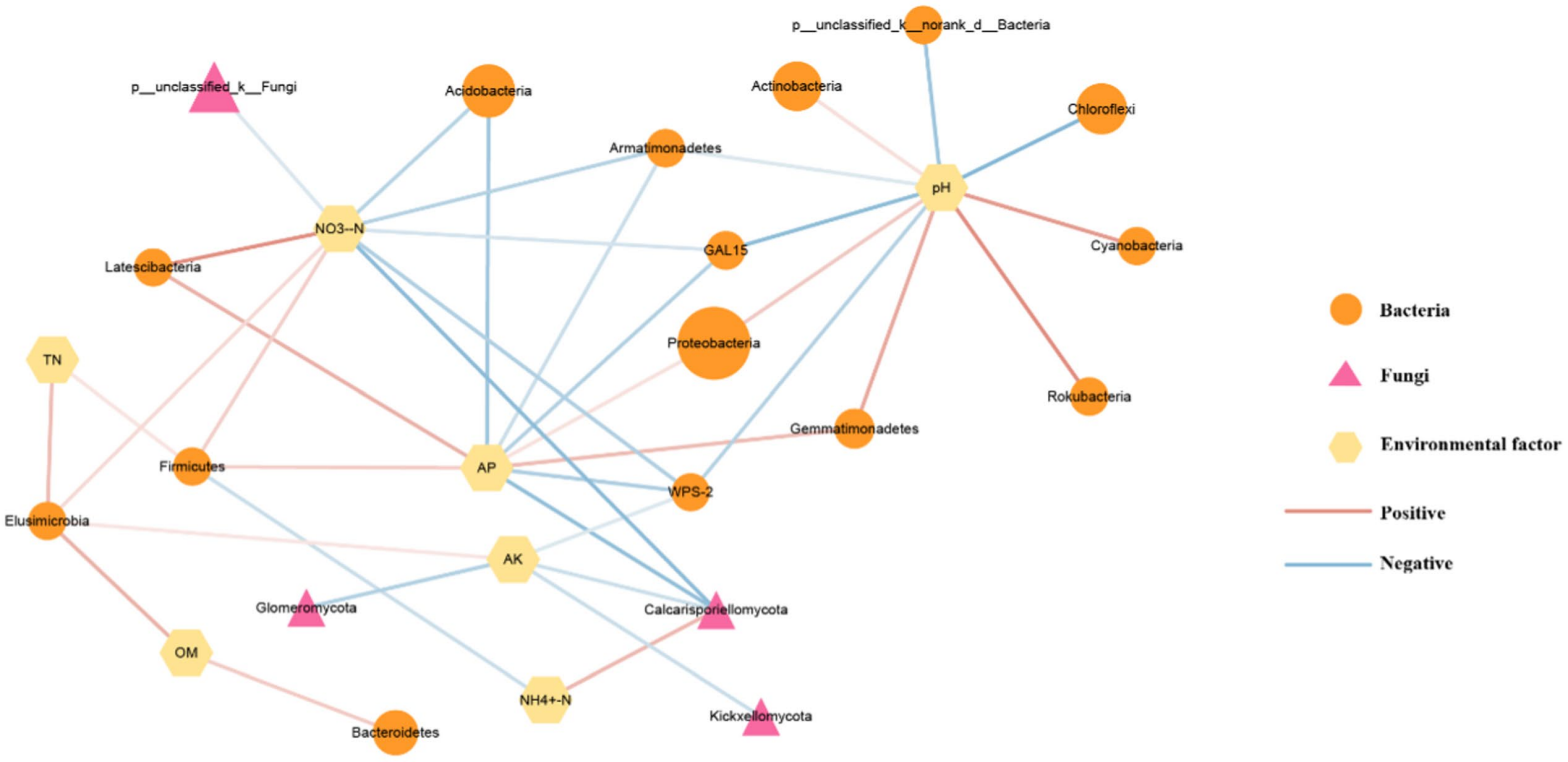
Correlation analysis of genusphylumate level microorganisms with environmental factors and functional prediction pathways. The graph size represents the relative abundance values of microorganisms, with the red line showing a positive correlation and the blue line showing a negative correlation.

In addition, correlation analysis was conducted on dominant microorganisms, environmental factors, and pathways related to carbon and nitrogen metabolism, and visualization mapping was carried out using Cytoscape (3.9.1) (Figure 8). The results indicate that environmental factors affect microorganisms at the gate level. Soil pH was positively correlated with the abundance of Proteobacteria, Gemmatimonadetes, Rokubacteria, Cyanobacteria, and Actinobacteria, and the abundance of Chloroflexi, Armatimonadetes, and GAL15 were negatively correlated. Our study showed a strong correlation between the soil pH value and the abundance of dominant bacteria in this experiment. AP was positively correlated with Gemmatimonadetes, Proteobacteria, Latescibacteria, and Firmicutes. It was negatively correlated with the abundance of Calcarisporiellomycota, WPS-2, GAL15, Armatimonadetes, and Acidobacteria. AK was positively correlated with the abundance of Elusimicrobia but negatively correlated with the abundance of spheromycota, Calcarisporiellomycota, and Kickxellomycota. OM, TN, and NH_4_^+^-N were associated with only two microorganisms. The carbon fixation pathways of prokaryotes predicted by PICRUSt2 function were positively correlated with pH, PAHs degradation, and nitrogen metabolism pathways were positively correlated with NH_4_^+^-N and negatively correlated with AK and NO_3_^−^-N.

## 4. Discussion

The use of inorganic fertilizer was reduced by straw returning and improved nutrient use efficiency and organic carbon (Akhtar et al., 2018; Wu et al., 2020; Li and Zhong, 2021; Wang et al., 2021; Li et al., 2022). Further, Su et al. (2020) and Huang et al. (2021) declared that straw returning improves soil functionality. In our study, no significant changes in the rhizosphere soil physicochemical properties and microbial community structure were noted at the seedling stage (Table S1) might be the incomplete decay of straw was due to the returned of straw for the short time in the field (Pimentel et al., 2019; Varanda et al., 2019). In the jointing stage, sugarcane grows vigorously, straw rot, s decomposes, and releases nutrient. The contents of TN, NO_3_^−^-N, SOC, and AK in the soil significantly increased due to the addition of straws. Straw returning to the field changes the soil chemical properties and improves soil quality (Wang et al., 2015). While regarding the contents of NO_3_^−^-N, AP, and AK were more in RR and ZZ compared with that of RZ and ZR. It is concluded that the composition of the similar cultivar straw was the same when returned to the field of the same cultivar., the inter-foliar structures of the same species were more similar, and thus the inter-foliar microbial interaction accelerated the process of degradation.

The decomposition of straw depends on soil microorganisms and thus is important for the soil environment (Zhao and Zhang, 2018; Zhao et al., 2019). Microorganisms play an important role in the decomposition, nutrient cycling, plant growth, and improving soil productivity (Hamayun et al., 2010; Alam et al., 2012; Nieto-Jacobo et al., 2017; Sánchez-Montesinos et al., 2019; Li et al., 2020). Diversity and composition of soil microbial communities are also related to soil quality and plant health (Berendsen et al., 2012; Bunemann et al., 2018). The diversity results (Table 3) showed that the richness and diversity of the microbial community of Z9 were higher than ROC22, and returning straws to the field increased the microbial diversity of the soil at the jointing stage Zeng et al., 2007; Bu et al., 2020. Generally, higher soil microbial diversity means that the relationship between microorganisms and the soil environment is more complex. The ecosystem’s stability was higher, and the ability to resist diseases was stronger (Chaparro et al., 2012; Niu et al., 2017; Yang et al., 2018). The cultivar Z9 and straw return improved the soil functionality, ecosystem, and disease resistance. Our analysis of microbial beta diversity PCoA (Figure 4) showed different treatment patterns. The rhizosphere bacteria of sugarcane were mainly composed of *Proteobacteria*, *Acidobacteria*, *Actinomycota*, *Chloroflexi*, and *Bacteroides*. These five phyla account for over 80% of the entire bacterial composition, consistent with the previous research results (Lian et al., 2019). The microbial composition of the two cultivars differed at the phylum level or the genus level (Figures 2, 3 and Supplementary Figure S2). Kong et al. (2020) studied the differences in microbial communities among different genotypes of maize. Generally, different plant species affect the structure of the soil microbial community by changing the secretion of metabolites and their interactions (Eviner et al., 2006; Edwards et al., 2015). These studies indicated that the change in plant genotypes between cultivars could affect the diversity and composition of microbial communities.

Returning of straw significantly influenced the composition of soil microbial community (Figures 2, 3). Many studies have shown that returning sugarcane straws to the field could affect the microbial community in the growing fields (Zhang C. et al., 2019; Zhang et al., 2021). In the samples of the jointing stage, the relative abundance of beneficial microorganisms *Gemmatimonadaceae, Trechispora, Streptomyces, Chaetomium,* etc., increased after straws returned to the field only *Pseudomonas* and *Aspergillus* increased significantly in the RR and ZZ groups (*p* < 0.01). *Gemmatimonadaceae* might be responsible for the organic removals to participate biological treatment process (Xu et al., 2017; Ding et al., 2018). *Trechispora* plays a significant role in wood degradation and the cycle of matter in the ecological system (Zhao and Zhao, 2021). *Streptomyces* is as efficient as a biofertilizer as a biocontrol and plant growth-promoting activity (Olanrewaju and Babalola, 2019). *Chaetomium* plays a crucial role in the degradation of many types of organic matter (Banerjee et al., 2016), because the straw was riched in organic matter. Li et al. (2020) isolated *Pseudomonas* spp. from the sugarcane rhizosphere and confirmed that it has plant growth-promoting properties and nitrogenase activity. *Aspergillus niger P85* could increase the fresh weight of corn (Yin et al., 2015). Shinde et al. (2020) synthesized magnesium hydroxide nanoparticles through *Aspergillus niger* filtrate, which could promote corn seed germination and seedling growth. In summary, both *Pseudomonas* and *Aspergillus* have been demonstrated to have potential plant yield-increasing effects. Therefore, the result could be linked to an increased yield of sugarcane (Figure 1). In conclusion, straw returning could improve soil microbial composition and increase the relative abundance of beneficial microorganisms.

Nutrients and soluble OM in crop straws might be released into the soil, forming a virtuous cycle with soil microorganisms (Wang et al., 2015). Luo et al. (2017) found that the increased activity of enzymes secreted by soil microorganisms might promote the decomposition of soil OM and meet the demand for microbial growth for carbon and nitrogen. Previous studies have found that Bacteroidetes member (*Bacteroides*) was the main microbial group involved in the decomposition of rice straw, including cellulose, hemicelluellulose, and chitin polysaccharides (Wegner and Liesack, 2016). Liu et al. (2012) found that Bacteroides were essential in degrading rice straw in rice soil. In this study, the experiment of straw returning sugarcane to the field of Z showed similar results. Compared with the Z group, the relative abundance of Bacteroidetes in the ZZ and ZR groups was increased, and OM also showed similar changes. Interestingly, the relative abundance of Bacteroidetes in the RR group decreased compared to the R group. Still, the OM content in RR increased compared with R. We noted that the relative abundance of Actinomyces in the RR group increased compared to that in the R group. Microbial-driven decomposition of plant residues is a component of carbon sequestration in terrestrial ecosystems. Actinomyces, one of the most widely distributed phyla of bacteria in soil, were known for their ability to degrade plant residues *in vitro* due to their high proportion of carbohydrate-active enzymes (CAZymes) and assist in improving nitrogen fixation and antibiotic production (Bao et al., 2021), and contribute to the global carbon cycle (Araujo et al., 2020). Therefore, Actinomyces competed with Bacteroidetes for organic matter. Present results showed that Actinomyces was positively correlated with pH. In our study, adding sugarcane straws of the same variety as planted sugarcane increased soil pH. Proteobacteria was related to the availability of nutrients and sensitivity to environmental changes (Fierer et al., 2012). Qi et al. (2018) showed that β- Proteobacteria was the most sensitive to changes in soil properties and was positively correlated with pH and AP. In this experiment, Acinetobacter was negatively correlated with AP and NO_3_^—^N, and Acidobacteria were identified as hypotrophic microorganisms that could only grow under very low nutrient conditions (Zeng et al., 2016). In Figure 8, Spheromycota, Calcarisporiellomycota, and Kickxellomycota were negatively correlated with AK. Liu et al. (2016) showed that some of the genera preferred to live in soils with specific fertility levels, and the addition of available nutrients would change the community composition of the species. Similar results were obtained in this experiment, and there was a negative correlation between the AK and the balloon bacteria. Thus, straw returning could alter the rhizosphere soil’s chemical characteristics and enhance soil quality and nutrient (C, N, P, and K) cycling in soil. It could affect soil microbial community structure and the abundance of dominant microbial communities and improve the microenvironment for plant growth.

## 5. Conclusion

The Z9 cultivar of sugarcane straw has more nutrients compared with ROC22, thereby improving soil functionality, ecosystem, and disease resistance compared with ROC22. The chemical and microbial community structure of sugarcane rhizosphere soil was significantly correlated with sugarcane straw retention and the species of straw. The microbial diversity of RR, ZZ, RZ, and ZR were significantly higher than R and Z after sugarcane straw retention. The microbial composition changed significantly, and individual species (*Phallus*) disappeared. The microbial communities of RZ and RR could be clearly distinguished from each other, as could ZZ and ZR. Since the diversity of RR and ZZ was higher than RZ and ZR, respectively, adding the same variety of sugarcane straw was more effective than adding different cultivars from the perspective of microorganisms. In addition, the two screened candidate microorganisms (*Pseudomonas* and *Aspergillus*.) may provide a reference for developing biological agents that promote sugarcane yield.

## Data availability statement

The datasets presented in this study can be found in online repositories. The names of the repository/repositories and accession number(s) can be found in the article/Supplementary material.

## Author contributions

RW, BH, MW and XQ: conceptualization and writing–original draft preparation. XQ and YS: methodology and formal analysis. JZ, TL, RW, and BH: investigation. RW, BC and BH: resources and supervision. MW and XQ: data curation. XQ, SA, BC, TL, KA, and RW: writing–review and editing. All authors contributed to the article and approved the submitted version.

## Funding

This work was supported in part by grants from Nanning Key R & D Plan (20201071), National Key R & D Program (2022YFD2301105-04), and the project on the safe utilization of agricultural land.

## Conflict of interest

The authors declare that the research was conducted in the absence of any commercial or financial relationships that could be construed as a potential conflict of interest.

## Publisher’s note

All claims expressed in this article are solely those of the authors and do not necessarily represent those of their affiliated organizations, or those of the publisher, the editors and the reviewers. Any product that may be evaluated in this article, or claim that may be made by its manufacturer, is not guaranteed or endorsed by the publisher.

## Supplementary material

The Supplementary material for this article can be found online at: https://www.frontiersin.org/articles/10.3389/fmicb.2023.1133973/full#supplementary-material

## Footnotes

^1^ www.majorbio.com

^2^ https://www.arb-silva.de/

^3^ www.majorbio.com
