# Supplementary material for: Sugarcane straw returning is an approaching technique for the improvement of rhizosphere soil functionality, microbial community, and yield of different sugarcane cultivars
Source: Front Microbiol. 2023 Mar 14;14:1133973. doi: 10.3389/fmicb.2023.1133973 (PMC10043380; doi:10.3389/fmicb.2023.1133973)
Supplement: Supplementary file 1 [file Data_Sheet_1.docx]

**Sugarcane straw returning is an approaching technique for the improvement of rhizosphere soil functionality, microbial community, and yield of different sugarcane cultivars**

Mengrong Wang^1†^, Xiaohang Qi^1†^, Yujie Shi^1^, Junyang Zhao^1^, Shakeel Ahmad^1^, Kashif Akhtar^1^, Baoshan Chen^1, 2^, Tengxiang Lian^3^, Bing He^2*^, Ronghui Wen^1*^

^1^State Key Laboratory for Conservation and Utilization of Subtropical Agro-bioresources, College of Life Science and Technology, Ministry and Province Co-sponsored Collaborative Innovation Center for Sugarcane and Sugar Industry, Guangxi Key Laboratory of Sugarcane Biology, Guangxi University, Nanning, China

^2^ Guangxi Key Laboratory of Agro-Environment and Agric-Products Safety, College of Agriculture, Guangxi University, Nanning, China

^3^The Key Laboratory of Plant Molecular Breeding of Guangdong Province, College of Agriculture, South China Agricultural University, Guangzhou, China

^*^Correspondence:

Bing He; Email: [hebing@gxu.edu.cn](mailto:hebing@gxu.edu.cn), Ronghui Wen; Email: [wenrh@gxu.edu.cn](mailto:wenrh@gxu.edu.cn)

^†^These authors share first authorship

Table S1 Soil characteristics of different treatments with straw and without straw returning at the seedling stage

| **Treatment** | **SOC**  **(g kg^-1^)** | **OM**  **(g kg^-1^)** | **TN**  **(g kg^-1^)** | **NH_4_^+^-N**  **(mg kg^-1^)** | **NO_3_^-^-N**  **(mg kg^-1^)** | **AP**  **(mg kg^-1^)** | **AK**  **(mg kg^-1^)** | **pH**  **(1:2.5)** |
| --- | --- | --- | --- | --- | --- | --- | --- | --- |
| **R** | 15.78±0.39a | 27.20±0.67a | 0.49±0.03a | 1.55±0.35a | 56.14±10.64a | 205.06±20.04ab | 159.78±7.00b | 5.70±0.21ab |
| **RR** | 15.19±0.48a | 26.19±0.83a | 0.47±0.08a | 1.36±0.19a | 53.92±6.76a | 146.91±10.27b | 245.92±8.87a | 6.24±0.20ab |
| **RZ** | 15.53±1.51a | 26.77±2.61a | 0.54±0.03a | 0.83±0.13a | 43.87±6.15a | 165.38±24.12ab | 173.99±6.36b | 5.60±0.31b |
| **Z** | 15.27±0.88a | 26.33±1.52a | 0.42±0.06a | 1.43±0.68a | 70.57±20.58a | 249.83±32.41a | 246.43±17.13a | 5.77±0.28ab |
| **ZZ** | 14.10±0.44a | 24.31±0.76a | 0.45±0.05a | 0.76±0.07a | 50.00±4.34a | 239.44±43.85ab | 263.02±21.16a | 6.41±0.16a |
| **ZR** | 15.97±0.84a | 27.54±1.46a | 0.54±0.09a | 0.55±0.07a | 57.59±4.94a | 154.69±15.33b | 238.65±10.58a | 5.94±0.11ab |

R: cultivar ROC22 without straw, RR: cultivar ROC22 plus with straw of ROC22; RZ: cultivar ROC22 plus with straw of Z9; Z: cultivar Z9 without straw; ZZ: cultivar Z9 plus with straw of Zhognzhe9; ZR: cultivar Z9 plus with straw of ROC22. OM=SOC × 1.724. Values are the means ± SE (n = 6).

Table S2 Soil characteristics of different treatments with straw at the mature stage

| **Treatment** | **SOC**  **(g kg^-1^)** | **OM**  **(g kg^-1^)** | **TN**  **(g kg^-1^)** | **NH_4_^+^-N**  **(mg kg^-1^)** | **NO_3_^-^-N**  **(mg kg^-1^)** | **AP**  **(mg kg^-1^)** | **AK**  **(mg kg^-1^)** | **pH**  **(1:2.5)** |
| --- | --- | --- | --- | --- | --- | --- | --- | --- |
| **R** | 18.20±0.18c | 31.4±0.31c | 1.60±0.01c | 6.07±0.22c | 13.46±1.79b | 76.46±5.31c | 79.98±0.66e | 6.43±0.01c |
| **RR** | 20.43±0.12b | 35.22±0.20b | 1.84±0.01b | 12.46±0.29a | 39.29±2.86a | 142.08±10.57b | 108.45±0.52c | 6.09±0.01f |
| **RZ** | 23.56±0.19a | 40.61±0.32a | 2.12±0.01a | 10.08±0.44b | 11.87±2.15b | 82.62±3.14c | 116.38±1.01b | 6.36±0.01d |
| **Z** | 15.75±0.16d | 27.16±0.28d | 1.41±0.00e | 12.20±0.52a | 12.94±2.03b | 81.38±7.32c | 97.22±17.13d | 7.00±0.02b |
| **ZZ** | 18.21±0.16c | 31.40±0.27c | 1.57±0.01d | 10.29±0.47b | 37.57±3.03a | 194.69±10.53a | 185.38±0.29a | 7.34±0.02a |
| **ZR** | 18.31±0.16c | 31.56±0.28c | 1.57±0.01d | 10.70±0.42b | 32.10±4.32a | 78.08±3.69c | 79.85±0.68e | 6.25±0.01e |

R: cultivar ROC22 without straw, RR: cultivar ROC22 plus with straw of ROC22; RZ: cultivar ROC22 plus with straw of Z9; Z: cultivar Z9 without straw; ZZ: cultivar Z9 plus with straw of Zhognzhe9; ZR: cultivar Z9 plus with straw of ROC22. OM=SOC × 1.724. Values are the means ± SE (n = 6).

Table S3 Alpha diversity of bacterial community and fungal community in different treatments with straw at the seedling stage

| **Treatment** | **Bacteria** | | | **Fungi** | | |
| --- | --- | --- | --- | --- | --- | --- |
|  | Chao1 | **Shannon** | **Coverage/%** | **Chao1** | **Shannon** | **Coverage/%** |
| **R** | 3197.49±97.14ab | 6.18±0.05a | 96.97±0.01ab | 838.56±50.29ab | 3.83±0.05a | 99.62±0.00a |
| **RR** | 3410.06±170.22ab | 6.28±0.15a | 96.74±0.00ab | 816.49±58.07ab | 3.64±0.17a | 99.64±0.00a |
| **RZ** | 3626.36±96.68a | 6.49±0.09a | 96.53±0.00b | 810.57±26.24ab | 4.00±0.14a | 99.68±0.00a |
| **Z** | 3107.06±160.32b | 6.14±0.14a | 97.12±0.00a | 556.89±60.86b | 2.37±0.22b | 99.78±0.00a |
| **ZZ** | 3457.66±122.94ab | 6.34±0.09a | 96.77±0.00ab | 939.85±60.18a | 3.77±0.25a | 99.65±0.00a |
| **ZR** | 3486.53±98.71ab | 6.37±0.11a | 96.76±0.00ab | 847.12±44.78ab | 3.63±0.27a | 99.70±0.00a |

Different letters in the same column the meant significant difference at P˃ 0.05.

Table S4 Alpha diversity of bacterial community and fungal community in different treatments with and without straw returning at the maturity stage

| **Treatment** | **Bacteria** | | | **Fungi** | | |
| --- | --- | --- | --- | --- | --- | --- |
|  | **Chao1** | **Shannon** | **Coverage/%** | **Chao1** | **Shannon** | **Coverage/%** |
| **R** | 3791.38±25.25c | 6.60±0.01c | 97.68±0.00a | 684.54±15.16c | 4.13±0.09bc | 99.77±0.00a |
| **RR** | 4198.78±9.29a | 6.68±0.01b | 97.37±0.00c | 791.31±10.65a | 3.93±0.06c | 99.70±0.00b |
| **RZ** | 4075.06±20.23b | 6.71±0.00a | 97.47±0.00b | 742.33±17.38ab | 4.05±0.04c | 99.71±0.00b |
| **Z** | 3621.88±36.31d | 6.50±0.01e | 96.03±0.00d | 727.43±19.38bc | 4.49±0.04a | 99.72±0.00ab |
| **ZZ** | 3710.93±29.58cd | 6.56±0.01d | 96.94±0.00e | 775.24±13.91ab | 3.55±0.08d | 99.61±0.02c |
| **ZR** | 3763.64±56.57c | 6.61±0.01c | 96.91±0.00d | 763.22±10.56ab | 4.29±0.06b | 99.64±0.00c |

Different letters in the same column meant significant difference at P˃0.05.


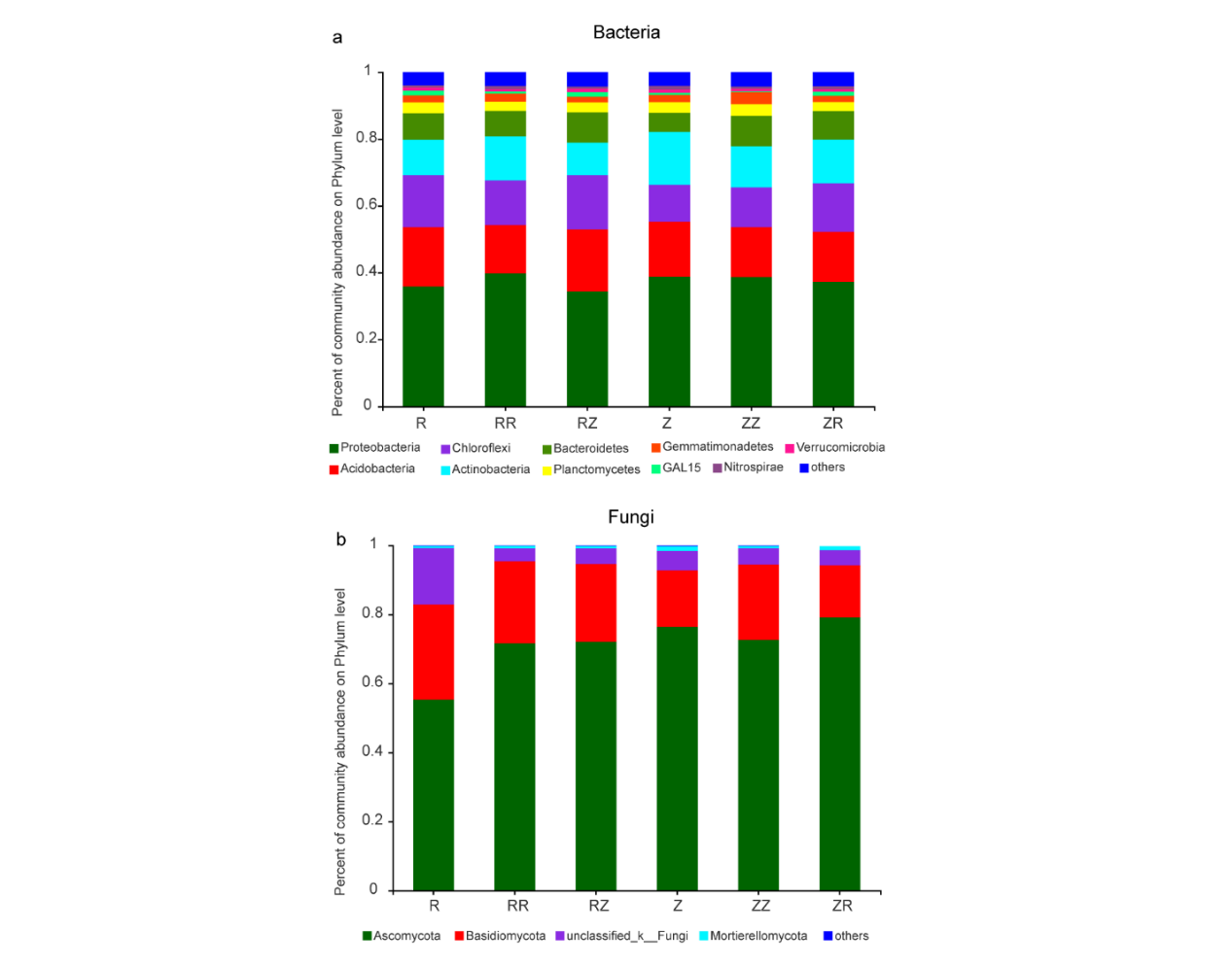


**Figure S 1** Relative abundance (% total reads) of the microbial community at phylum level of different sugarcane cultivars


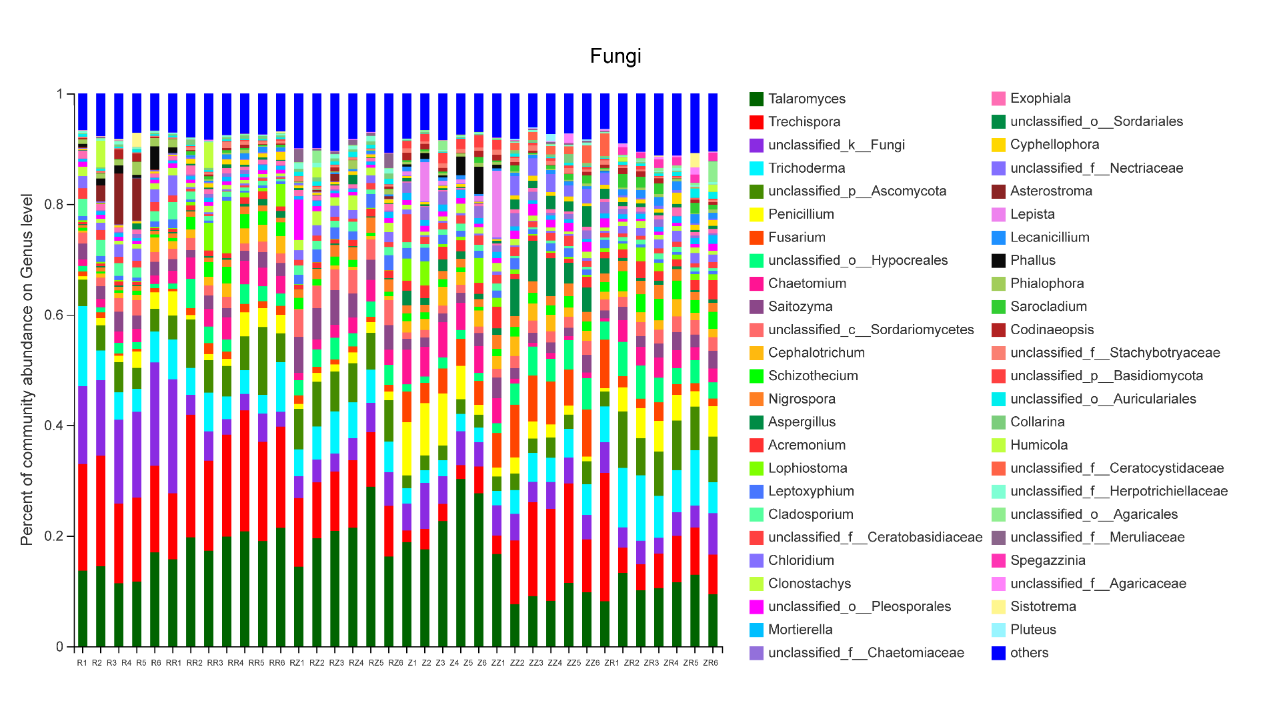


**Figure S 2** Relative abundance (% total reads) of fungal community at genus level of different sugarcane cultivars


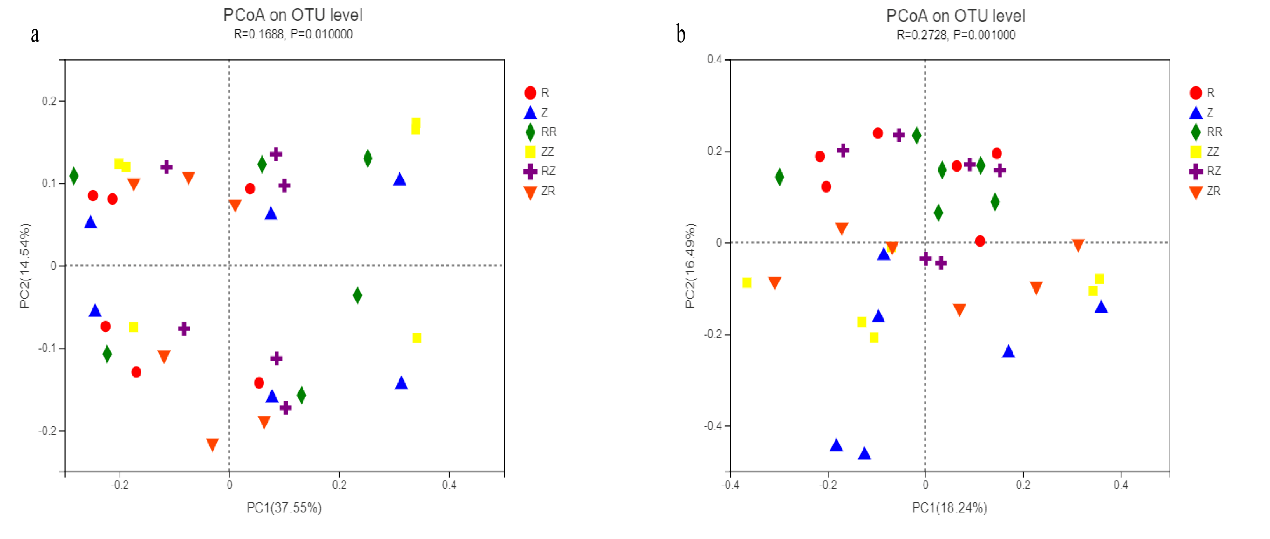


**Figure S 3** Principal Coordinates analysis (PCoA) of bacterial (a) and fungal (b) community structure at the seeding stage of different sugarcane cultivars
